# Astrocytes as a target for therapeutic strategies in epilepsy: current insights

**DOI:** 10.3389/fnmol.2023.1183775

**Published:** 2023-07-31

**Authors:** Nihan Çarçak, Filiz Onat, Evgenia Sitnikova

**Affiliations:** ^1^Department of Pharmacology, Faculty of Pharmacy, Istanbul University, Istanbul, Turkey; ^2^Institute of Health Sciences, Department of Neuroscience, Acibadem Mehmet Ali Aydinlar University, Istanbul, Turkey; ^3^Department of Medical Pharmacology, Faculty of Medicine, Acibadem Mehmet Ali Aydinlar University, Istanbul, Turkey; ^4^Institute of Higher Nervous Activity and Neurophysiology of Russian Academy of Sciences, Moscow, Russia

**Keywords:** temporal lobe epilepsy, absence epilepsy, epileptogenesis, gliotransmission, neuroglia, astrocytes, astrocyte-targeting therapy, ictogenesis

## Abstract

Astrocytes are specialized non-neuronal glial cells of the central nervous system, contributing to neuronal excitability and synaptic transmission (gliotransmission). Astrocytes play a key roles in epileptogenesis and seizure generation. Epilepsy, as a chronic disorder characterized by neuronal hyperexcitation and hypersynchronization, is accompanied by substantial disturbances of glial cells and impairment of astrocytic functions and neuronal signaling. Anti-seizure drugs that provide symptomatic control of seizures primarily target neural activity. In epileptic patients with inadequate control of seizures with available anti-seizure drugs, novel therapeutic candidates are needed. These candidates should treat epilepsy with anti-epileptogenic and disease-modifying effects. Evidence from human and animal studies shows that astrocytes have value for developing new anti-seizure and anti-epileptogenic drugs. In this review, we present the key functions of astrocytes contributing to neuronal hyperexcitability and synaptic activity following an etiology-based approach. We analyze the role of astrocytes in both development (epileptogenesis) and generation of seizures (ictogenesis). Several promising new strategies that attempted to modify astroglial functions for treating epilepsy are being developed: (1) selective targeting of glia-related molecular mechanisms of glutamate transport; (2) modulation of tonic GABA release from astrocytes; (3) gliotransmission; (4) targeting the astrocytic Kir4.1-BDNF system; (5) astrocytic Na^+^/K^+^/ATPase activity; (6) targeting DNA hypo- or hypermethylation of candidate genes in astrocytes; (7) targeting astrocytic gap junction regulators; (8) targeting astrocytic adenosine kinase (the major adenosine-metabolizing enzyme); and (9) targeting microglia-astrocyte communication and inflammatory pathways. Novel disease-modifying therapeutic strategies have now been developed, such as astroglia-targeted gene therapy with a broad spectrum of genetic constructs to target astroglial cells.

## 1. Introduction

Glial cells, also known as neuroglia, are non-neuronal cells of the central and peripheral nervous system (Kettenmann and Ransom, 2004a; Kettenmann and Verkhratsky, 2008; Verkhratsky and Butt, 2013). There is a great diversity of neuroglia in respect to their origin, localization, morphology, physiological, and functional properties. Traditionally, four types of glial cell have been recognized in adults: (1) Astrocytes, (2) Microglia, (3) oligodendrocytes, and (4) their progenitors, neuron–glial antigen 2 (NG2) glia (Kettenmann and Ransom, 2004b; Kettenmann and Verkhratsky, 2008; Verkhratsky and Butt, 2013). Astrocytes, the most abundant type of glial cells in the brain, are very heterogeneous cells that provide physical and metabolic support for neurons, regulate the extracellular environment of neurons, are involved in blood-flow regulation, and have a close association with the microvasculature (Jabs et al., 2008; Lanerolle et al., 2010; Verhoog et al., 2020; Borodinova et al., 2021). Microglia are involved in first-line innate immunity to the central nervous system (Carson et al., 2007; Soulet and Rivest, 2008; Yang et al., 2010; Saijo and Glass, 2011). Oligodendrocytes synthesize myelin and wrap it around axons in the central nervous system and Schwann cells—in the peripheral nervous system. Damage or loss of the myelin sheath around axons is common in many neurological disorders (Kurosinski and Götz, 2002; Barres, 2008). The NG2-glia has been identified as a progenitor for mature oligodendrocytes within the oligodendrocyte lineage. NG2-glia keep generating mature myelinating oligodendrocytes throughout lifetime. Even though these cells have been the subject of a considerable amount of research, the function of NG2-glia in epileptogenesis has not been established yet (Dimou and Gallo, 2015).

**Figure 1:**
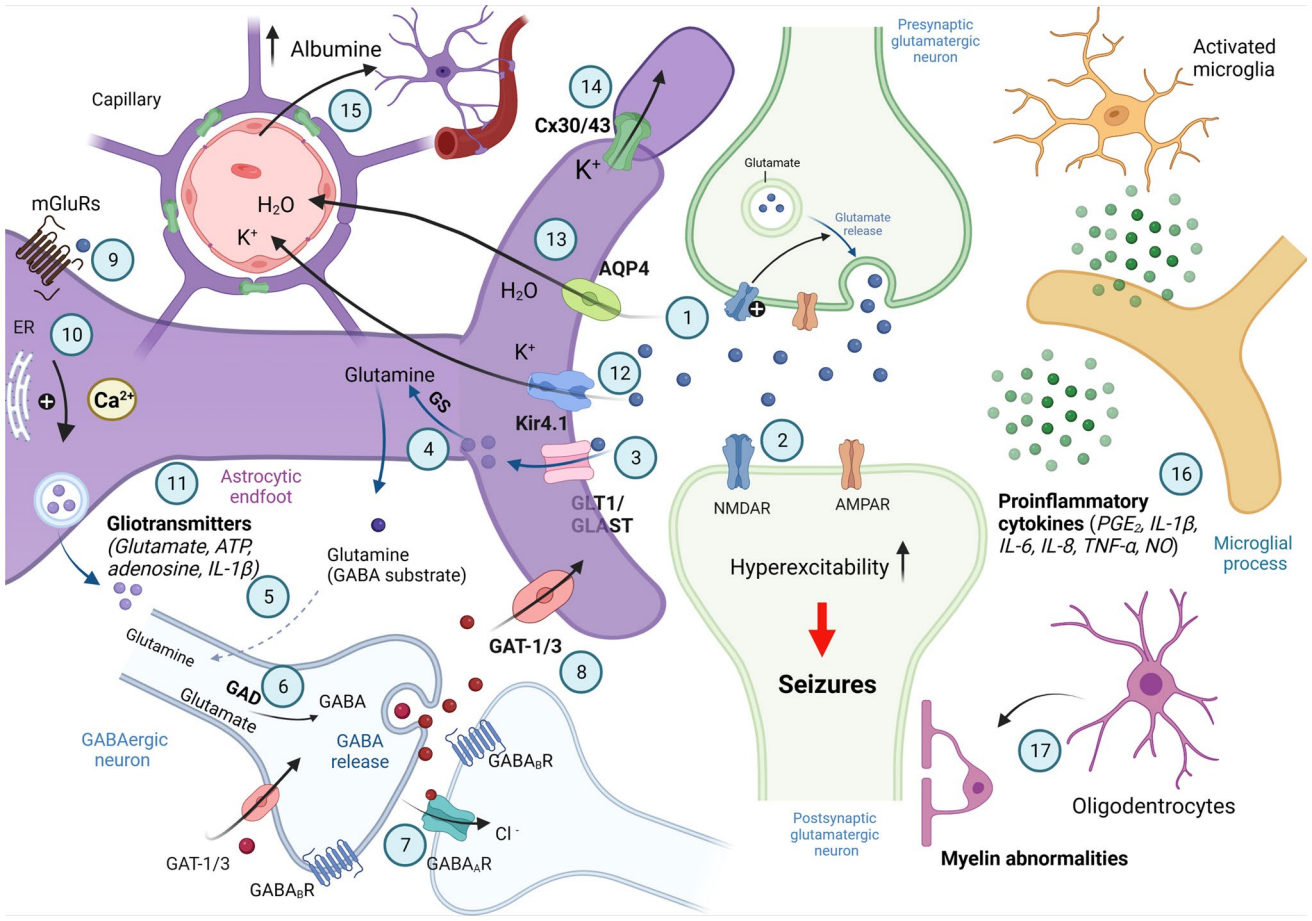
Involvement of astrocytes in synaptic excitability. Schematic illustration of signaling between astrocytes and neurons associated with active control of astrocytes of neuronal activity; synaptic neurotransmission is shown by two active synapses: one glutamatergic (light green) and the nother synapse is GABAergic (bottom); parts of reactive astrocytes shown as activated microglia and oligodentrocytes. Generated action potentials in the pre-synaptic glutamatergic neuron results in the excocytotic synaptic release of the neurotransmitter glutamate (1). Glutamate activates AMPA and NMDA receptors in the post-synaptic glutamatergicneuron, and excitatory postsynaptic potential is generated by influx of Na^+^ and Ca^2+^ (2). Glutamate is taken up into astrocytes by the EAAT-1 and EAAT-2 transporters localized on the astrocytic membranes (GLAST and GLT-1 in rat; 3) and converted to glutamine by glutamine synthetase (GS; 4). Glutamine is taken up by GABAergic neurons (5) where it is converted to glutamate by glutaminase and then to GABA by glutamic acid decarboxylase (GAD; 6). When released from presynaptic vesicles into the synaptic cleft, GABA diffuses across and binds to postsynaptic GABA_A_ and GABA_B_ receptors. It may also bind to presynaptic GABA_B_ receptors. GABA binds to specific recognition sites on the GABA_A_ receptor, and this triggers a conformational change leading to opening of the intrinsic anion channel allowing chloride ions to flow through into the cell, resulting in hyperpolarization of the neuron (7). GABA is removed from the synaptic cleft into surrounding astrocytes or the presynaptic terminal by GABA transporters (GAT; 8). Neurotransmitters released by depolarized neurons activate the astrocytic G protein-coupled receptors (GPCRs; 9). The GPCRs activate the phospholipase C/inositol 1,4,5-triphosphate (PLC/IP3)-mediated pathway for the release of Ca^2+^ from intracellular calcium stores, such as the endoplasmatic reticulum (ER), resulting in an increase in intracelluar Ca^2+^ (10), intracellular Ca^2+^ elevations in astrocytes stimulate gliotransmitter release (11) that can influence neuronal excitability. K^+^ released from neurons enters astrocytes via inward rectifying K^+^ channels (Kir 4.1) and is distributed into capillaries (12). The astrocytic water channel Aquaporin-4 (AQP4) mediates the flow of water between the extracellular space and the blood to maintain osmotic balance (13). Astrocytes are connected to each other via gap junction channels composed of connexin 30 (Cx30) and connexin 43 (Cx43), which mediates spatial buffering of K^+^ ions (14). Associated with the breakdown of the blood–brain barrier or with changes in blood flow and extravasation of molecules such as albumin microglia are activated (15). Activated microglia release proinflammatory cytokines involved in neuroinflammation and reactive gliosis (16). Oligodendrocytes and abnormalities in myelination of postsynaptic neuron are also involved in control of synaptic excitability (17; Created with BioRender.com).

Glial cells and neurons closely interact with each other (Kurosinski and Götz, 2002; Magistretti, 2006; Barres, 2008; Allen and Barres, 2009; Xu et al., 2016). According to the concept of the tripartite synapse, perisynaptic oligodendrocytes and synaptically associated astrocytes are thought to be integral elements involved in synaptic functions (Araque et al., 1999; Figure 1). A new morpho-functional concept of the “active milieu” of the brain refers to a close interaction between compartments of neurons, astrocytes, oligodendrocytes, microglia, blood vessels, the extracellular space, and the extracellular matrix (Semyanov and Verkhratsky, 2021). Neuron–glia interactions within the active milieu are more complex than those posited in the concept of the multipartite synapse. Dysfunction of neuron–glia interactions has been recognized and well documented in various neurological diseases (Verkhratsky and Butt, 2013), in particular, in neurodegenerative diseases (Lobsiger and Cleveland, 2007; Malva et al., 2007; Kim et al., 2020), brain tumors (Yang et al., 2010; Saijo and Glass, 2011), tuberous sclerosis complex (Wong, 2019), encephalopathy (Hamid et al., 2018), and epilepsy (Pollen and Trachtenberg, 1970; Glötzner, 1973; Bordey and Sontheimer, 1998; O’Connor et al., 1998; Heinemann et al., 2000; Perez Velazquez and Carlen, 2000; Steinhäuser and Seifert, 2002; D’Ambrosio, 2004; Tian et al., 2005; Binder and Steinhäuser, 2006; Li et al., 2007; Jabs et al., 2008; Wetherington et al., 2008; Lanerolle et al., 2010; Seifert et al., 2010; Aronica et al., 2012; Devinsky et al., 2013; Vezzani et al., 2013; Verhoog et al., 2020; Grigorovsky et al., 2021; Heuser et al., 2021; Lentini et al., 2021; Sano et al., 2021; Wang et al., 2021).

Although all glial cell types play a pivotal role in normal brain function, in this review we will focus on astrocytes, which are key homeostatic regulators in the central nervous system and play important roles in the pathophysiology of epilepsy.

According to the definition of the International League Against Epilepsy (ILAE) Commission, epilepsy is a disorder of the brain characterized by an enduring predisposition to generate epileptic seizures, and by the neurobiologic, cognitive, psychological, and social consequences of this condition (Fisher et al., 2005, 2017a,b). In addition, an epileptic seizure is a transient occurrence of signs and/or symptoms due to abnormal excessive or synchronous neuronal activity in the brain. Epilepsy has long been considered to result from a disorder of neuronal cells and neuronal circuits. This is mainly due to excessive neuronal firing (hyperexcitation) and abnormally strong synchronization in widespread neuronal populations (hypersynchronization). This idea was formulated by Ransom and Blumenfeld (2007): “*Modern neuroscience has demonstrated that seizures are the result of abnormal, synchronized paroxysms of electrical activity in a population of neurons (an epileptogenic focus) that are able to rapidly recruit other parts of the brain to share in its rhythmic, self-sustaining electrical discharge*.” With this information in mind, abnormal activity of neurons has long been acknowledged as the primary source of epileptic activity. However, it has become evident that epilepsy, as a chronic disorder of the central nervous system, has been accompanied by dysfunction of glial cells and by impairment of communications between neurons and neighboring glial cells. The role of glial cells in epilepsy has attracted increasing attention over the last few decades, and many studies have shown that all types of glial cells are involved in epileptogenesis (Steinhäuser and Seifert, 2002; D’Ambrosio, 2004; Tian et al., 2005; Binder and Steinhäuser, 2006; Li et al., 2007; Jabs et al., 2008; Seifert et al., 2010; Steinhäuser et al., 2012; Grigorovsky et al., 2021; Heuser et al., 2021; Sano et al., 2021). A deeper understanding of the relationship between glial cells and epilepsy may provide insights into developing novel anti-seizure and anti-epileptogenic therapies (Brennan and Henshall, 2020; Peterson and Binder, 2020; Verhoog et al., 2020).

The clinical diagnosis of epilepsy relies on a detailed medical history and results of a thorough diagnostic evaluation (Fisher et al., 2017a; Devinsky et al., 2018). The classification of epilepsies considers clinical signs (semiology) of seizures as well as the underlying cause (etiology) of seizures. This review follows an etiology-based approach to epilepsy classification and extends this approach to astrocyte-related mechanisms. Considering the fact that changes in astrocytic functions may account for clinical signs of epilepsy, this review aims to bridge the gap between clinical and basic studies of epileptic disorders.

## 2. The etiologic categories of epilepsy

Major advances in molecular biology, cell biology, biochemistry, gene/protein functions contribute to a better understanding of underlying causes (or the etiologic basis) of epilepsy. The etiological classification of epilepsies was proposed based on epilepsies with genetic origins, epilepsies with structural/metabolic causes, and epilepsies of unknown cause (Berg et al., 2010). Then, in 2017, the International League Against Epilepsy (ILAE) Commission on Classification and Terminology defined six etiological categories: structural/metabolic/immune/infectious, genetic, and unknown (Fisher et al., 2017b; Scheffer et al., 2017). In this review, we will address structural/metabolic/immune/infectious and genetic etiological concepts. Nevertheless, the unknown etilogical category has not been addressed in this review because the concept has not been well defined.

1. Structural/metabolic/infectious/immune: Conceptually, there is a distinct other structural or metabolic condition or disease that has been demonstrated to be associated with a substantially increased risk of developing epilepsy in appropriately designed studies. Structural lesions of course include acquired disorders, such as stroke, trauma, and infection. They may also be of genetic origin (e.g., tuberous sclerosis, many malformations of cortical development); however, as we currently understand it, there is a separate disorder interposed between the genetic defect and the epilepsy. An immune etiology is related to autoimmune-mediated central nervous system inflammation against neuronal antigens such as receptors and synaptic proteins (Lancaster and Dalmau, 2012; Scheffer et al., 2017).
2. Genetic: The concept of genetic epilepsy is that epilepsy is, as best as understood, the direct result of a known or presumed genetic defect(s) in which seizures are the core symptom of the disorder. The knowledge regarding the genetic contributions may derive from specific molecular genetic studies that have been well replicated and even become the basis of diagnostic tests (e.g., SCN1A and Dravet syndrome) or the evidence for a central role of a genetic component may come from appropriately designed family studies. Designation of the fundamental nature of the disorder as genetic does not exclude the possibility that environmental factors (outside the individual) may contribute to the expression of disease.
3. Unknown cause: Unknown is meant to be viewed neutrally and to designate that the nature of the underlying cause is as yet unknown; it may have a fundamental genetic defect at its core or it may be the consequence of a separate as yet unrecognized disorder.

Astrocytes are the most abundant cells in the central nervous system (Araque et al., 1999; Kettenmann and Ransom, 2004a; Volterra and Meldolesi, 2005; Allen and Barres, 2009; Kaur and Ling, 2013; Verhoog et al., 2020; Borodinova et al., 2021). Astrocytes play a crucial role in neuronal development and the anatomical and functional organization of neural circuits. More specifically, astrocytes are involved in neurogenesis and synaptogenesis, in maintaining extracellular ion balance, in controlling neuronal excitability, and in controlling the permeability of the blood–brain barrier (Araque et al., 1999; Angulo et al., 2004; Wallraff et al., 2006; Allen, 2013; Harada et al., 2016; Verhoog et al., 2020). They regulate synaptic transmission between the presynaptic and postsynaptic neurons, thus modulating synaptic transmission as the third part of the tripartite synapse (Araque et al., 1999; Xu et al., 2016). Astrocytes communicate with each other via gap junctions that permit direct cell–cell transfer of ions and small molecules (i.e., metabolites, catabolites, and second messengers). Upon the gap junctions, the calcium wave propagates from astrocyte to astrocyte (Figure 1). In addition, the membranes of astrocytes contain specific transport proteins involved in neurotransmitter reuptake from synaptic clefts (glutamate, GABA, and glycine). Apart from controlling the ionic environment of the neuropil and controlling the supply of neurotransmitters to synapses, astrocytes can directly activate neuronal receptors by releasing chemical transmitters called “gliotransmitters.” These include peptides, chemokines, and cytokines (Halassa and Haydon, 2010; Seifert et al., 2010; Devinsky et al., 2013; Vezzani et al., 2013; Harada et al., 2016; Verhoog et al., 2020; Figure 1). Halassa et al. (2007) introduced the concept of the “astrocyte activation spectrum,” in 2007, suggesting that enhanced gliotransmission might contribute to epilepsy, and reduced gliotransmission—to schizophrenia. Astrocytes effectively modulate synaptic transmission between neurons and the extracellular environment through the release of gliotransmitters (such as glutamate, d-serine, and ATP). In normal conditions, astrocytes are in a constant state in the neuronal circuits. However, insults to the nervous system in various pathological contidions shift their function to an “activated state.” This process is called “*reactive astrogliosis*,” and it is a key feature of the epileptic focus in both humans and experimental conditions (Binder and Steinhäuser, 2021). The multiple mechanisms by which astrocytes contribute to epilepsy will be examined in further detail in the next sections of this review, following an etiology-based approach.

## 3. The role of astrocytes in structural/metabolic/infectious/immune epilepsy

Structural/metabolic/infectious/immune epilepsies (Table 1) are caused by external/environmental/internal factors (such as perinatal/infantile causes, cerebral trauma, cerebral infection, hippocampal sclerosis, cerebral tumor, cerebrovascular disorder, cerebral immunologic disorder, degenerative, and other neurologic conditions). Temporal lobe epilepsy (TLE) is the most common epilepsy syndrome of acquired epilepsy that received the closest attention and is characterized by the prominent dysfunction of astrocytes (Ransom and Blumenfeld, 2007; Wetherington et al., 2008; Verhoog et al., 2020). Studies from TLE patients and related animal models have demonstrated the role of astrocytes in the onset and development of TLE. According to these studies, astrocyte dysfunction contributes to neuronal hyperexcitation, neurotoxicity, and epileptogenesis, or the seizure spread (Steinhäuser et al., 2012). Reactive astrogliosis in response to the initial insult, is frequently prominent and almost always coexists with hippocampal sclerosis, which is the most common histopathological finding in TLE (Twible et al., 2021). Proposed mechanisms of astrocyte involvement in structural/metabolic/infectious/immune epilepsy particularly in TLE will be summarized below.

**Table 1:** Involvement of astrocytes in different etiologic categories of epilepsy (in patients with epilepsy and in animal models of epilepsy).

Etiologic category of epilepsy	Cell type	Functioning	References
Genetic	Astrocytes	Malfunction of the astrocytic GABA, transporter GAT-1	Crunelli et al. (2011)
		Lower expression of glutamate transporter (GLUT-1), increased expression of GFAP, and increased glutamine-glutamate cycle	Dutuit et al. (2002); Melø et al. (2006); Çavdar et al. (2019)
		Gap junction proteins (Cx30/43)	Çavdar et al. (2021)
		Induction of IL-1β in GAERS rats genetic absence epilepsy rats from strasbourg	Akin et al. (2011)
		In tuberous sclerosis: astrogliosis, changes in morphology, increased GFAP	Wong (2019)
		In focal cortical dysplasia: upregulation of astroglial metabotropic receptors mGluRs	Devinsky et al. (2013)
Structural/metabolic/immune/infectious	Astrocytes	Activation of astrocytes	Tian et al. (2005); Devinsky et al. (2013); Sano et al., (2021)
		Glutamate: upregulation of astroglial metabotropic receptors mGluRs	Lanerolle et al. (2010); Devinsky et al. (2013); Peterson and Binder (2020)
		Glutamate: alterations in astroglial ionotropic receptor GluR1	Binder and Steinhäuser (2006)
		Glutamate: dysregulation of astrocytic glutamate transporters, GLT-1, and GLAST	Binder and Steinhäuser (2006); Peterson and Binder (2020)
		Potassium: downregulation of astroglial Kir4.1, and imbalance of normal extracellular potassium homeostasis	Ransom and Blumenfeld (2007); Kinboshi et al. (2020)
		Gliosis: proliferation of astroglia	Losi et al. (2012); Zamanian et al. (2012); Binder and Steinhäuser (2006)
		Electrolyte imbalance and osmolality: reduced expression of astroglial AQP4 and swelling of astrocytes	Binder et al. (2012); Murphy et al. (2017)
		BBB. Dysfunction of the BBB lead to epileptogenesis via astroglial TGF-bR	Ivens et al. (2007); Heinemann et al. (2012)
		Gap junctional contacts: enhanced astrocyte-astrocyte coupling mediated by proteins connexin-43 and connexin-30	Wallraff et al. (2006); Nagy and Rash (2000); Walrave et al. (2020)
		Gap junctional contacts: selective inhibition of astrocytic gap junctions suppress synchronization over astrocytic syncytium providing an anti-epileptic effect	Volnova et al. (2022)

GABA, gamma-aminobutyric acid; GAERS rats, genetic absence epilepsy rats from strasbourg; GFAP, glial-fibrillary acidic protein; mGluRs, metabotropic glutamate receptors; GluR1, ionotrotic glutamate receptor; GLT-1, astroglial glutamate transporter 1; GLAST, astroglial glutamate aspartate transporter; Kir4.1, inwardly rectifying K+ channels; AQP4, aquaporin-4 water channels; BBB, blood–brain barrier; TGF-bR, astroglial growth factor b receptor; TNF-α, tumor necrosis factor; IL-1β, interleukin 1β; and IL-6, interleukin 6.

### 3.1. Glutamate

In neurons, glutamate is the principal excitatory neurotransmitter, playing a key role in the initiation and spread of epileptic activity. Both neurons and astrocytes are capable of releasing glutamate (neurotransmitter and gliotransmitter, respectively; Harada et al., 2016). Astrocytes are known to be involved in many glutamate-mediated activities: glutamate synthesis, reuptake of released glutamate, and disposal of excess glutamate (Hertz and Zielke, 2004; Halassa and Haydon, 2010; Xu et al., 2016). As a part of the tripartite synapse, astrocytes can sense neural activity *via* metabotropic and ionotropic glutamate receptors. Once glutamate binds to an astrocytic metabotropic glutamate receptors (mGluRs), the astrocyte’s second messenger systems are activated. Activation of mGluR3 affects cAMP accumulation, and activation of mGluR5 leads to an increase in intracellular Ca^2+^ (Binder and Steinhäuser, 2006). The initiation and the propagation of Ca^2+^ waves within the astrocytic network may induce glutamate release from astrocytes mediated by Ca^2+^-dependent ion channels (Araque et al., 1999; Devinsky et al., 2013; Figure 1). Astrocytic glutamate may directly excite neighboring neurons and contribute to seizure generation (Kang et al., 2005). Electron-microscopic and immunohistochemical inspection of sclerotic hippocampal tissue from TLE patients revealed expression of mGluR2/3, mGluR4, mGluR5, and mGluR8 in reactive astrocytes, suggesting an involvement of these receptors in gliosis (see Binder and Steinhäuser, 2006; Lanerolle et al., 2010). Astrocytic mGluRs are upregulated in patients with mesial temporal sclerosis and with focal cortical dysplasia as a compensatory response to prevent seizures (see Lanerolle et al., 2010; Devinsky et al., 2013). Peterson and Binder (2020) published a review in 2020, in which they summarized the current knowledge regarding the astrocytic mGluRs in human TLE and animal models.

Secondly, astrocytic ionotropic glutamate receptors are also involved in the processes of epileptogenesis, as it has been shown in many studies (reviewed in Binder and Steinhäuser, 2006). Changes in the GluR1 and GluR2 subunits of AMPA receptors have been found in sclerotic hippocampi in patients with TLE. More specifically, an elevated flip-to-flop mRNA ratio of the GluR1 splice variant in reactive astrocytes may associate with an increase in responsiveness of these astrocytes to glutamate (see Binder and Steinhäuser, 2006; Lanerolle et al., 2010).

Glutamate transporters are expressed by several types of neurons and glia, but astrocytes are primarily responsible for glutamate uptake (Peterson and Binder, 2020). Glutamate transporters rapidly remove excess glutamate from the extrasynaptic space, resulting in termination of synaptic events and preventing excitotoxic injury of neurons (reviewed by Ransom and Blumenfeld, 2007; Verhoog et al., 2020). Astrocytes express Na^+^-dependent glutamate transporters, such as glutamate transporter-1 (GLT-1) and glutamate aspartate transporter (GLAST; Figure 1). GLT-1 is responsible for ∼90% of glutamate uptake in the adult dorsal forebrain and is crucial for the maintenance of low extracellular glutamate to permit efficient synaptic transmission (reviewed by Peterson and Binder, 2020). Downregulation of GLT-1 protein levels has been observed in the kainic acid-induced seizure mouse model (i.e., a pharmacological model of TLE). GLT-1 levels have been shown to have decreased levels in the hippocampus of TLE patients with hippocampal sclerosis (Peterson and Binder, 2020). Sha et al. (2017) demonstrated that upregulation of GLT-1 protein level had antiepileptic effects in the kainic acid model of TLE and in knockout mouse model of tuberous sclerosis complex (T_SC_1^GFAP^CKO mice). These authors showed that the substance 17AAG prevented degradation of GLT-1 by disrupting the association between Hsp90β and GLT-1. A reduction in the astrocytic glutamate transporter GLT-1 could have therapeutic potential for the treatment of epilepsy. Supporting this hypothesis, a novel mechanism of action on astrocytic GLT-1 has been described for gabapentin as an anti-seizure drug (Yoshizumi et al., 2012). *In vitro* and *in vivo* results confirmed that gabapentin increases extracellular glutamate by astroglial glutamate transporter-mediated mechanisms (Yoshizumi et al., 2012; Suto et al., 2014).

In addition to GLT-1 and GLAST, the cystine/glutamate exchanger (xCT; encoded by SLC7A11), which transports a cystine inside while exchanging for glutamate. This contributes to astrocyte glutamate release (Cuellar-Santoyo et al., 2023). The SLC7A11/xCT is an essential sodium-dependent glutamate transporter that is crucial for determining ambient glutamatergic concentration in the central nervous system (Augustin et al., 2007). Its expression occurs mainly in astrocytes (Ottestad-Hansen et al., 2018). SLC7A11/xCTprotein expression was elevated in about 50% of patient tumors, associated with faster growth, peritumoral glutamate excitotoxicity, seizures, and decreased survival. The use of sulfasalazine, an xCT inhibitor decreased the glutamate release from the tumor (Robert et al., 2015). Supporting this clinical data, sulfasalazine reduced the NMDA-induced current by 66.8% in mouse hippocampus slices (Koh et al., 2022) and decreased neuronal hyperexcitability *in vitro* (Alcoreza et al., 2019). Also, intraperitoneal administration of sulfasalazine significantly reduced seizure burden in beta-1 integrin knockout mice, a model of astrogliosis-mediated epilepsy *in vivo* (Alcoreza et al., 2022). These data suggest that selective targeting of glia-associated molecular mechanisms of glutamate transport may be an effective pharmacological strategy to ameliorate tumor-associated seizures and develop novel anti-seizure drugs.

### 3.2. Gamma-aminobutyric acid

Tonic GABA release from astroctyes mediated by the reverse action of glial GABA transporter (GAT) subtypes, maintains the excitation/inhibition (E/I) balance within the central nervous system. The activity of the glutamate transporter triggers the reversal of GABA transporters through increasing astrocytic Na⁺ concentration. Then, GABA causes tonic inhibition in a network activity-dependent way. This is an example of an *in situ* negative feedback mechanism by which astrocytes convert the glutamatergic excitation to GABA-ergic inhibition for modifying the excitability of neurons (Héja et al., 2012). Studies in experimental models of TLE reported that GABA released by reactive astrocytes from Bestrophin-1 channels strongly contributes to the compensatory shift of E/I balance in epileptic hippocampi. The tonic release of GABA from astrocytes is one of the key mechanisms by which astrocytes control epileptic seizures (Crunelli et al., 2011; Muller et al., 2020; Goisis et al., 2022). By modulating tonic GABA release from astrocytes, it might be possible to target epileptic seizures and other neurological disorders (Muller et al., 2020; Pandit et al., 2020).

### 3.3. Potassium

Astrocytes control neuronal hyperexcitability by regulating the release of K^+^ ions from neurons into the extracellular space during action potentials. During neuronal hyperactivity, the extracellular [K^+^] may increase from a resting level of around 3 mM to a ceiling of 10–12 mM and be taken up by astrocytes (Binder and Steinhäuser, 2006). The K^+^ reuptake process is mediated by the inwardly rectifying K^+^ channels (Kir channels) and other astrocyte K^+^ transporters. Among the Kir channels (Kir1-Kir7), the subtype Kir4.1 has been most thoroughly investigated in brain astrocytes. Structural epilepsy with hippocampal sclerosis is known to be accompanied by a downregulation of Kir4.1 and a derangement of normal extracellular potassium homeostasis (Ransom and Blumenfeld, 2007). Kitaura et al. (2018) reported impairment of the potassium buffering system in astrocytes in mesial temporal lobe epilepsy. They suggested that a decreased activity of Kir4.1 channels in astrocytes induced hyperexcitability in the subiculum in sclerotic hippocampus.

Recently, the role of astrocytic Kir4.1 channels in epileptogenesis has been evaluated by Kinboshi et al. (2020) who concluded that reduction of Kir4.1 channels resulted in elevation of the level of extracellular K^+^ and of the level of synaptic glutamate, and this facilitated the expression of brain-derived neurotrophic factor (BDNF) in astrocytes. BDNF is a key mediator of epileptogenesis that mediates synaptic plasticity, neuronal sprouting, neurogenesis and reactive astrogliosis (Scharfman, 2005; Ohno et al., 2018). BDNF is expressed in both pyramidal neurons and astrocytes (Saha et al., 2006). BDNF expression was upregulated in both neurons and astrocytes during epileptogenesis (Thomas et al., 2016). These findings support the concept that inhibition of Kir4.1 channels, which occurs under certain epileptic conditions, facilitates BDNF expression in astrocytes. The astrocytic Kir4.1-BDNF system has the potential to serve as a novel target for the treatment of epilepsy (Kinboshi et al., 2020). On top of that, growing evidence suggest the potential impact of astroglial functions through other neuromodulatory inputs such as endocannabinoid signaling (Eraso-Pichot et al., 2023).

Astrocytic K^+^ buffering mediated by Na^+^/K^+^/ATPase is also critically involved in seizure susceptibility. In a model of focal cortical dysplasia (FCD) in rats, Zhao et al. (2022) found that *“the astrocytic Na^+^/K^+^/ATPase-mediated buffering of K+ contributes to activity-dependent inhibition of hyperexcitable pyramidal neurons during seizure and can attenuate neocortical seizures*.*”* In this study, optogenetic activation of astrocytes exhibited anti-seizure effect on neocortical seizures following cortical kainic acid injection, in a Na^+^/K^+^/ATPase dependent manner (Zhao et al., 2022). The authors concluded that optogenetic control of astrocytic Na^+^/K^+^/ATPase activity can be considered as a promising therapeutic approach for the treatment of epilepsy. These experiments demonstrate that astrocytes can effectively control both seizure initiation and seizure spread.

### 3.4. Electrolyte imbalance and osmolality

Astrocytes are known to form the glymphatic system, or “pseudo-lymphatic” perivascular network, distributed throughout the brain (Reddy and van der Werf, 2020). The glymphatic system contributes to the transport of nutrients and signaling molecules into the brain parenchyma, meanwhile, promoting the clearance of proteins and interstitial waste solutes out of the brain (Natale et al., 2021). The glymphatic pathway is known to be impaired in different neurological pathologies, mainly neurodegenerative diseases (Alzheimer’s disease), neurovascular disorders (hemorrhagic and ischemic), and other acute degenerative processes (normal pressure hydrocephalus or traumatic brain injury; Natale et al., 2021). In regard to the glymphatic system, changes in the aquaporin-4 (AQP4) water channels have been less clear, considering the following works: Eid et al. (2005), Binder et al. (2012), Peixoto-Santos et al. (2017), and Tice et al. (2020). These channels are located primarily on the astrocytic end-feet and are actively engaged in the circulation of interstitial and cerebrospinal fluids through the arterial perivascular spaces (Tice et al., 2020). The glial AQP4 plays an important role in modulating brain excitability and epilepsy, as reviewed by Binder and co-authors (Binder et al., 2012). These authors concluded: “*while many questions remain unanswered, the available data indicate that AQP4 and its molecular partners may represent important new therapeutic targets*.”

Another important issue is volume regulation in astrocytes. Cells may be swollen or shrunken by osmotic stress and return to their original volume even in the continued presence of the osmotic stress (Murphy et al., 2017). In animal models of structural epilepsy (i.e., induced by injections of pentilentetrazol, PTZ or electrical stimulation of hippocampus) astrocyte swelling was observed through specific targeting of AQP4 or Kir4.1 (see Murphy et al., 2017). Genetic mutations in the gene KCNJ10, which encodes Kir4.1, is known to cause seizures in humans (see Boni et al., 2020).

### 3.5. Blood–brain barrier

Astrocytes are key players in buffering neurons from the harmful effects of the bloodstream, such as oxidative stress and toxic chemicals. Astrocytes also form part of the blood–brain barrier (BBB) as the outer wall of the brain capillary endothelium is enclosed by pericytes and astrocyte end feet, anatomically assembled to guarantee barrier functions (Giannoni et al., 2018). The integrity of the BBB is impaired during epileptogenesis (Friedman et al., 2009). The primary dysfunction of the BBB is a hallmark of brain insults and brain inflammation, in which microglia play a major role (see below), and astrocytes are also responsible for some epileptogenic processes (Heinemann et al., 2012). Ivens et al. (2007) in a rat model confirmed the role of astrocytes in epileptogenesis after BBB opening and before development of epileptiform activity. In particular, they showed that “e*pileptogenesis in the BBB-disrupted brain seems to be mediated by exposure of the brain cortex to serum albumin, mediated via its action on brain astrocytes*” (cited by Friedman et al., 2009). In addition, they also reported (Ivens et al., 2007) a cascade of events during this window period of epileptogenesis, and identified transforming growth factor b receptor (TGF-bR) as a key player in the cellular response resulting in abnormal accumulation of extracellular potassium and consequent activation of NMDA receptors. Therefore, amelioration of neural injury caused by disruption of BBB may be achieved via targeting the TGF-bRs.

Perivascular astrocytes are central to the structural and functional changes of the microvasculature in epilepsy through their combination of the roles in maintaining the BBB integrity and release of pro-inflammatory cytokines (including vascular endothelial growth factor, IL-1β, HMGB1, and TNF), chemokines such as C-C motif ligand 2 (CCL2) as well as downstream effectors, which also activate receptors on pericytes and endothelial cells of microvessels (Giannoni et al., 2018; Yamanaka et al., 2021a). As a consequence of this neuroinflammatory state, peripheral immune cells, including monocytes, B and T cells, and neutrophils infiltrate into the brain and activate resident microglia (Yamanaka et al., 2021b). Experimental data demonstrated that infiltration of peripheral cells into the brain promote brain inflammation and exacerbate neuronal damage after status epilepticus (SE; Varvel et al., 2016). An increased expression of CCL2 which is an especially potent chemoattractant for cells of monocytic lineage (including macrophages, monocytes, and microglia) was observed in the astrocytes of the epileptic human and rat brain (Bozzi and Caleo, 2016; Broekaart et al., 2018). Supporting that, blocking the blood-derived cell invasion into the brain by knocking out CCR2, a receptor for CCL2 that affects monoycte migration, attenuated neuronal damage and suppress seizures in SE models (Varvel et al., 2016) and by knocking out of T and B cells leads to a decline in seizure activity in kainite model of (TLE) (Zattoni et al., 2011). Human data also revealed that seizure frequency was correlated with the number of infiltrating peripherally activated monocytes, but not microglia in intractable pediatric epilepsy (Xu et al., 2018). Experimental and human data supports the interplay among the astrocytes, microglia, and peripheral monocytes that can affect epileptogenesis and may lead to progression of epilepsy via increased leakage of the BBB. Neverthless, current understanding of this crosstalk between the microglia, blood-derived infiltrating cells, and astrocytes is still limited and relative importance of infiltrated cell types in epileptogenic process remain to be determined. Further studies needed to better understand the importance of the role of microglia and blood-derived-infiltrating monocytes and (perivascular) macrophages in the crosstalk with astrocytes and whether specific manipulations of this interaction can modify or prevent epileptogenesis. Rossi et al. (2017) have demonstrated that subacute treatment with gabapentin initiated immediately after the initial insult during the early latency period, successfully reduced reactive gliosis, macrophage infiltration and increased convulsive threshold in the long-term in post-SE model of pilocarpine. These results suggest that subsequent blood-derived macrophage interaction with resident microglia and astrocytes probably has an additional role on the microglial and astroglial activation during the latency period in acquired epilepsies.

### 3.6. Gap junctional contacts

Intercellular contacts between astrocytes have the nature of gap junctions (Nagy and Rash, 2000; Steinhäuser et al., 2012; Stephan et al., 2021). Astrocytes (and oligodendrocytes) form a non-synaptic communication called a glial syncytium through the connexin-based gap junctions in the membranes of neighboring cells. This allow ions, metabolites, and currents to pass (Stephan et al., 2021). In the adult brain, hippocampal astrocytes express two major gap junction proteins: connexin-43 (Cx43) and connexin-30 (Cx30; Nagy and Rash, 2000). As early as in 2006, Wallraff and co-authors (Wallraff et al., 2006) examined electrophysiological properties of the hippocampus in transgenic mice with conditional deletion of Cx43 in astrocytes and additional unrestricted deletion of Cx30. It appeared that hippocampal slices from mice with coupling-deficient astrocytes displayed a reduced threshold for the generation of epileptiform events. At the same time, an unexpectedly large capacity for K^+^ clearance in these mice indicated that gap junction-dependent processes only partially account for K^+^ buffering in the hippocampus. Connexin-based gap junctions have a complex and ambiguous role in epilepsy (Walrave et al., 2020). It is well documented that Cx43 mRNA levels, protein expression, phosphorylation state, distribution and/or functional coupling are altered in human epileptic tissue and experimental models (Walrave et al., 2020). In addition, Cx30/Cx43-based gap junctions are necessary for trafficking nutrients to neurons and initiating of astrocytic Ca^2+^ waves and hyper-synchronization, thereby supporting a proconvulsant effect.

Recently, Volnova et al. (2022) examined the role of gap junctions in epileptic activity *in vitro* using a 4-aminopyridin pharmacological model. These authors suggested a scheme in which astrocytes interconnected by gap junctions disperse the excessive release of neuronal K^+^. The astrocytes absorb K^+^ through the Kir1.4 channels and releasing it into the bloodstream. Moreover, they showed that a premedication with carbenoxolone (a gap junction blocker) in a 4-aminopyridin model of epilepsy decreased the latency period of seizure, decreased total seizure duration, and prevented the development of the status epilepticus (Volnova et al., 2022). Pharmacotherapy for epilepsy could also target astrocytic gap junction regulators. Levetiracetam as a clinically approved anti-seizure drug also exerts anti-inflammatory effects on glia, including reconstitution of gap junction coupling in cultured astrocytes (Bedner et al., 2015).

### 3.7. Adenosine

Adenosine functions as a neuromodulator functions as an endogenous anticonvulsant in the brain, and also act as a seizure terminator (Dragunow, 1991). Adenosine has an inhibitory effect on neuronal activity mediated by the activation of G_i_-protein coupled adenosine receptors express on both neuronal and glial cells (Fredholm, 2012). Astrocytes play an important role in regulating adenosine levels. Nucleoside transporters expressed on the astrocyte membrane remove the adenosine form the extracellular space and stop adenosine-mediated signaling through the metobolising enzyme adenosine kinase (ADK), which is mainly localized to astrocytes (Boison et al., 2010). ADK overexpression as a general response to astroglial activation (Boison and Aronica, 2015) is considered a neuropathological feature of temporal lobe epilepsy (Aronica et al., 2011), focal cortical dysplasia (Guo et al., 2023), and contribute to the epileptogenic process itself (Gouder et al., 2004). In line with those observations in humans, ADK inhibitors showed an antiepileptogenic property in intrahippocampal kainic acid mouse model of temporal lobe epilepsy (Sandau et al., 2019). These findings support that an adenosine augmentation approach by using either adenosine receptor agonists or ADK inhibitors might be a promising treatment strategy for drug-resistant epilepsy.

### 3.8. DNA methylation

Maladaptive epigenetic changes, which include methylation of DNA and acetylation of histones play a functional role in the development of epilepsy and its progression. Methylation hypothesis for epileptogenesis suggested that seizures can induce epigenetic chromatin modifications, thereby aggravating the epileptogenic condition (Kobow and Blumcke, 2011). Supporting that, experimental and clinical studies provided some evidence epigenetic processes particularly increased DNA methyltransferase activity and abnormal DNA methylation contribute to seizures and epileptogenesis (Williams-Karnesky et al., 2013; Martins-Ferreira et al., 2022). As mentioned in the previous section, dysregulation of adenosine homeostasis due to overexpression of the key adenosine-metabolizing enzyme ADK leads to exacerbation of epilepsy and a general response to astrocyte activation (Boison and Aronica, 2015). Adenosine tone can directly modulate DNA methylation *in vivo* and thereby exert additional epigenetic effects via biochemical interference with the transmethylation pathway (Williams-Karnesky et al., 2013). Through enhanced DNA methylation, maladaptive increases in ADK expression in astrocytes drive the epileptogenic process (Williams-Karnesky et al., 2013). Therefore therapeutic adenosine augmentation or ADK inhibition preventing maladaptive DNA methylation can be considered as a rational approach to prevent epileptogenesis.

In a recent study, Boni et al. (2020) showed that DNA methylation can bidirectionally modulate transcription of the gene KCNJ10 representing *“a targetable molecular mechanism for the restoring astroglial Kir4.1 expression after central nervous system (CNS) insult.”* Therefore, DNA hypo- or hypermethylation of candidate genes in astrocytes might be a promising genetic targets for therapeutic intervention of epileptogenesis (Williams-Karnesky et al., 2013). DNA methylation could potentially alter astrocyte reactivity and inflammatory status through controlling the signaling transducer and activator of transcription 3 (STAT3) pathway, along with other signaling pathways (O'Callaghan et al., 2014; Neal and Richardson, 2018).

The field of epigenetics is still relatively new and particularly few studies have examined astrocyte-specific DNA methylation in neurological diseases. There are still significant gaps in knowledge regarding how epigenetic mechanisms can influence astrocytes. Despite the fact that it is obvious that astrocytes in the adult brain express the numerous proteins required for the various epigenetic pathways including DNA methylation, histone modifications, and miRNAs, suggesting that these factors could be potentially involved in the astrocyte response to inflammation and epileptogenic process.

### 3.9. Astrogliosis

Reactive astrogliosis is a type of astrocyte response to an initial insult such as traumatic brain injury, stroke, status epilepticus, and viral infections. These activated astrocytes then contribute to the epileptogenic process (Binder and Steinhäuser, 2021; Purnell et al., 2023). Studies in humans and experimental models demonstrate that there is a bidirectional relationship between astrocytes and neuroinflammation: reactive astrocytes release cytokines such as inter-leukin (IL)-1b, IL-6, tumor necrosis factor (TNF)-a, transforming growth factor beta (TGF)-b, and chemokines, such as monocyte chemoattractant protein-1(MCP-1), chemokine, C-C motif, ligand 2 (CCL2; Aronica et al., 2012) that amplify epileptogenic inflammatory signaling. As above mentioned, astrocytes by releasing cytokines play a significant role in peripheral inflammation induced neuroinflammation by activating neighboring cells (pericytes and endothelial cells of microvessels) and amplify the local, initial innate immune response further; and also modify BBB permeability resulting in the recruitment of immune cells from the blood circulation into the neural tissue. This leads to an amplification of the initial innate immune reaction by activating microglia, and thus supporting an adaptive immune response (Farina et al., 2007; Riazi et al., 2010). On the other hand, inflammatory cytokine and chemokine release from astrocytes can also be triggered by a variety of cytokines, chemokines, and danger signals such as high mobility group box 1 (HMGB-1). The latter can be released by reactive microglia or the microvasculature after an initial insult (Maroso et al., 2010; Eyo et al., 2017). Microglia-astrocyte crosstalk is vital for normal neuronal function (Jha et al., 2019; Matejuk and Ransohoff, 2020). Microglia typically respond first to any pathological insult in the CNS, which is followed by astrocytic activation. Molecular mechanisms of microglia-astrocyte crosstalk in the CNS health and disease attract major attention (Jha et al., 2019; Matejuk and Ransohoff, 2020; Yang et al., 2020). Molecular therapy might target epilepsy-related aberrant communications between microglia and astrocytes.

A nuclear protein, HMGB-1, received particular attention, because it has an important role in models of non-infectious inflammation, such as autoimmunity, cancer, trauma, and ischemia reperfusion injury (Klune et al., 2008). HMGB-1 is known to be released from damaged neurons after, for example, traumatic brain injuries and serves as a prognostic biomarker and therapeutic target in patients (Parker et al., 2017). Animal models of acute and chronic seizures have shown that HMGB-1 receptors are expressed after experimental seizures (Maroso et al., 2010; Rossi et al., 2017) and HMGB-1/Toll-like receptor-4 (TLR4) signaling plays a role in generating and perpetuating seizures and might be targeted to attain anticonvulsant effects in epilepsies (Maroso et al., 2010). Supporting this, inhibiting the inflammatory cytokine HMGB-1 in CNS by using monoclonal antibodies (Fu et al., 2017) and in the periphery by systemic application of glycyrrhizin (Rosciszewski et al., 2019) reduced inflammation and has demonstrated therapeutic value in numerous electrical and chemical animal models of TLE, as well as in human tissue (Zhao et al., 2017). Additionally, inflachromene had an anti-seizure potential as a small molecule HMGB-1 inhibitor, as was shown in different animal models of epilepsy (Dai et al., 2023). HMGB-1 may also be involved in the upregulation of P-glycoproteins during status epilepticus, which is linked to drug resistance (Xie et al., 2017). These findings suggest that HMGB-1 released after the initial insult acting on astrocytes and microglia activates TLRs and its downstream signaling pathways which are proposed as key early steps in epileptogenesis (Rosciszewski et al., 2019). Thus, either HMGB-1 blockage or TLR antagonist molecules would be able to reduce neuroinflammation and may be a novel target for developing anti-seizure drugs especially for drug-resistant epilepsies or anti-epileptogenic drugs.

## 4. The role of astrocytes in genetic generalized epilepsies

The term “genetic generalized epilepsies (GGEs)” describes all types of generalized epilepsies with a presumed genetic basis. Idiopathic generalized epilepsies (IGEs) can be recognized as a distinct subgroup of GGEs comprised of the following syndromes: childhood absence epilepsy, juvenile absence epilepsy, juvenile myoclonic epilepsy, and epilepsy with generalized tonic–clonic seizures alone (Hirsch et al., 2022). GGEs also include epilepsy with myoclonic-atonic seizures, epilepsy with eyelid myoclonia, epilepsy with myoclonic absences, and myoclonic epilepsy in infancy (MEI or Dravet Syndrome). Latter have a genetic basis and are associated with developmental and epileptic encephalopathy (Hirsch et al., 2022).

Among IGEs, the most knowledge about glia-related mechanisms was gained from animal models of generalized non-convulsive absence epilepsy (Crunelli and Leresche, 2002). Genetic rodent models of generalized epilepsy with spontaneous spike-and-wave discharges (SWDs) on the EEG exhibit defined periods of seizure onset followed by seizure progression. Absence seizure frequency increases in an age dependent manner (Crunelli and Leresche, 2002). Astrocytes get involved in disturbances of thalamic GABA-ergic and glutamatergic neurotransmission (Crunelli et al., 2020).

The astrocytic GABA transporter has been reported to be impaired in genetic models of absence seizures (Crunelli et al., 2011). More specifically, an enhanced GABA-ergic tonic inhibition in thalamic neurons, which is required for absence seizure generation, is associated with a malfunction of the astrocytic thalamic GABA transporter-1 (GAT-1). The neuro-glial relationships relating to glutamatergic transmission are also affected in an age-dependent manner in experimental absence epilepsy models. Lower expression of glutamate transporters (GLT1 and GLAST) in cortical primary astrocytes obtained from Genetic Absence Epilepsy Rats from Strasbourg (GAERS) suggests an impairment of turnover of transporter proteins, which leads to lower levels of glutamate uptake in the cortex and thalamus of GAERS (Dutuit et al., 2000; Touret et al., 2007). Another observation is the increased expression of glial fibrillary acidic protein (GFAP) as the first sign of reactive astrocytes in genetic absence epilepsy rat models. This suggests the presence and involvement of reactive astrocytes in the epileptogenesis of non-convulsive seizures (Dutuit et al., 2000; Çavdar et al., 2019). In addition, an increased cycling of glutamate and glutamine between astrocytes and glutamatergic neurons in the cortex of GAERS indicates a dysregulation of astrocyte-neuron interactions in the thalamo-cortical loop (Melø et al., 2006, 2007; Touret et al., 2007; Onat et al., 2013; Gobbo et al., 2021).

Astrocytic gap junctions formed by connexin (Cx) 30 and Cx43 (Figure 1) are also likely to play a role the pathogenesis of absence epilepsy. Astrocytic gap junction protein expressions increased in the thalamo-cortical circuitry in rat models of absence epilepsy (Gobbo et al., 2021). *In vivo*, gap junction blocker carbeoxolone reduced absence seizures (Gigout et al., 2006; Crunelli et al., 2015; Çavdar et al., 2019).

An astrocyte-modulating agent, ONO-2506 (arundic acid), appeared to have specific anti-epileptic properties in a mouse model of non-convulsive epilpesy (Yamamura et al., 2013). ONO-2506 was discovered by Ono Pharmaceutical Co as an agent inhibiting astrocytic S-100β (i.e., acidic calcium-binding protein produced mainly by astrocytes). ONO-2506 inhibited absence epilepsy in a genetic mouse model of absence epilepsy [Cacna1atm2Nobs/tm2Nobs mice, in which the conditional calcium channel (Cacna1a) gene was knocked-down], but it did not affect maximal electroshock seizures (MES) or pentylenetetrazol-induced seizures (PTZ; Yamamura et al., 2013). In fact, ONO-2506 did not affect convulsive seizures in traditional models of epilepsy, but markedly inhibited epileptic phenomena in a genetic epilepsy mouse model. These data suggest that enhancing gliotransmitter release might be the way to go for a novel glial-targeting drugs for non-convulsive seizures (Onat, 2013; İdrizoğlu et al., 2016).

**Figure 2:**
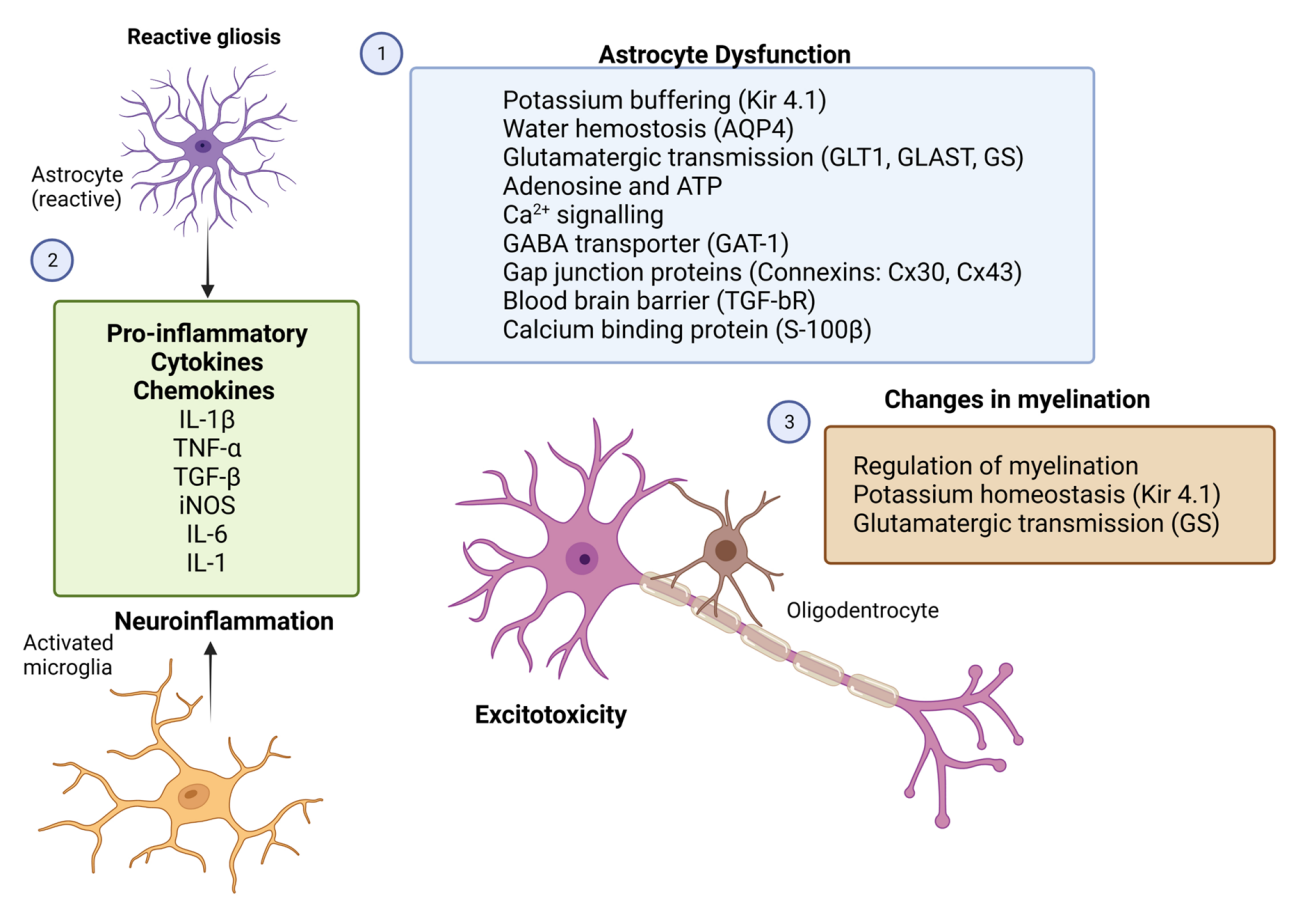
Potential therapeutic targets for glial cells in epilepsy. Rectangles with different colors represent the potential molecular candidates that can target astrocytes (1,2), microglia (2) and oligodendrocytes (3). GFAP, glial-fibrillary acidic protein; GLT-1, astroglial glutamate transporter 1; GLAST, astroglial glutamate aspartate transporter; GS, glutamine synthase; İNOS, inducible nitric oxide; Kir4.1, inwardly rectifying K+ channels; AQP4, aquaporin-4 water channels; BBB, blood–brain barrier; TGF-bR, astroglial growth factor b receptor; TNF-α, tumor necrosis factor; IL-1β, interleukin 1β; IL-1, interleukin 1; and IL-6, interleukin 6 (Created with BioRender.com).

The astrocytic interleukin-mediated mechanism has been identified, again, in a genetic model of absence seizures. In GAERS, the focal epileptic zone in the somatosensory cortex is characterized by induction of interleukin (IL)-1β in activated astrocytes (Akin et al., 2011). The authors of this study suggested a specific anti-inflammatory approach by blocking IL-1β biosynthesis, which may be helpful for managing this non-convulsive form of epilepsy. Cytokine (TNF-α, IL-1β) expression was associated with the development of absence seizures. The expression of these cytokines tended to increase before the seizure onset in GAERS and WAG/Rij (Wistar Albino Glaxo rats from Rijswijk) rat models. This might be considered as a form of neuroprotection toward adaptive mechanisms linked to generalized seizure activity (Akin et al., 2011; van Luijtelaar et al., 2012). This is also in line with results showing that a specific anti-inflammatory approach, the interruption of the IL-6 signaling pathway through administration of tocilizumab, a neutralizing humanized monoclonal antibody to the IL-6 receptor reduced the development of absence seizures and depressive-like comorbidity in the GAERS model (Leo et al., 2020). In agreement with that, IL-6 and IL-8 levels in the cerebrospinal fluid fluid have indeed been linked to human childhood absence epilepsy (Billiau et al., 2007) and valproic acid treatment lowers IL-6 serum levels in children with tonic–clonic generalized seizures (Steinborn et al., 2014). In order to treat this non-convulsive form of epilepsy and its associated neuropsychiatric comorbidities, targeting cytokines and chemokines may provide a new avenue for the development of targeted anti-epileptogenic therapies (Figure 2).

So far, evidence for linking the astroglial fine-tuning of the extracellular ion and transmitter homeostasis to absence seizures is still lacking. Yet, their involvement in controlling such network excitability is very likely, just as it is for other types of epileptiform activity. Therefore, more in-depths investigation is needed to determine the involvement of astroglial Kir4.1 channels in the occurrence of SWDs. Combining pharmacological models of absence epilepsy with cell-specific conditional knock-outs of these channels may shed new light on the subject.

Growing evidence also suggests that astrocytes are involved in the pathology of epileptic encephalopathies. Dravet syndrome (DS)or myoclonic epilepsy in infancy (MEI) is a rare, genetic epileptic encephalopathy which is linked to *de novo* loss of function mutations of the SCNA1 gene encoding for the α subunit of Nav1.1 voltage-dependent sodium channels (Claes et al., 2001). In experimental models of DS, during the development of seizures, changes in GABA_A_ extrasynaptic receptor composition and GABA tonic currents have been recently shown to occur in parallel with an increase in microglia and astrocyte reactivity (Satta et al., 2021; Goisis et al., 2022). Moreover, GAT-1 is expressed in the nerve endings of GABAergic interneurons and astrocytes. A functional deficit in thalamic astrocytes may result in enhanced tonic inhibition in SLC6A1 variants associated with epilepsy (Mermer et al., 2022). Astrocyte reactivity was observed after the onset of spontaneous seizures in a mouse model of SCN8A encephalopathy. SCN8A encephalopathy is caused primarily by gain-of-function mutations in the neuronal sodium channel (Nav1.6) and causes severe, early-onset seizures. Reactive astrocytes have reduced Kir4.1 channel currents and reduced expression of GS protein, suggesting an impairment in K^+^ ion uptake and glutamate homeostasis leading to the increase in seizure frequency in this mouse model (Thompson et al., 2022).

## 5. Conclusion and future directions: astroglial targets for treatment of epilepsy

Epilepsy is a chronic disorder of the central nervous system characterized not only by neuronal hyperexcitation and hypersynchronization, but also by substantial disturbances in large populations of glial cells and by impairment of neuron-glial interactions. A better understanding of glial pro-epileptic and epileptogenic mechanisms is helpful for the development of novel anti-seizure, anti-epileptogenic, and/or neuroprotective therapies. Astrocytes are the most prominent cell population within the glia, and they are deeply involved in controlling of neuronal excitability *via* releasing gliotransmitters, glutamate-dependent mechanisms (through astroglial metabotropic and ionotropic glutamate receptors), potassium-dependent mechanisms (through astroglial Kir4.1 and changes in gap junctional contacts), in controlling electrolyte balance and osmolality (through astroglial AQP4), in controlling the blood–brain barrier (through astroglial TGF-bR), regulation of blood flow and energy metabolism, and in supporting synapse formation (through the production of NG2; Figure 2). Since astrocytes involved in the variety of physiologic functions, it is not unexpected that astrocytic dysfunction has been linked to many CNS pathologies including epilepsy. Data from experimental models and clinical studies indicate that astrocytes are both contribute to development of epilepsy (epileptogenesis) and seizure generation and recurrence (ictogenesis; Vezzani et al., 2022; Purnell et al., 2023).

In this review, we have presented the key functions of astrocytes contributing to neuronal hyperexcitability and synaptic activity following an etiology-based approach. In this part, we will analyze the role of astrocytes in both development (epileptogenesis) and generation of seizures (ictogenesis) and comment on the therapeutic strategies that attempted to modify astoglial responses in each time point of the epilepsy.

### 5.1. Role of astrocytes in epileptogenesis and anti-epileptogenic strategies

Epileptogenesis is a complex gradual process whereby healthy brain is transformed into an epileptic state occurring over months to years. It begins with a brain-damaging insult that triggers a cascade of molecular and cellular changes that lead to spontaneous recurrent seizures (Pitkänen and Lukasiuk, 2009; Maguire, 2016). Maguire (2016) considered the three stages of epileptogenesis: (1) the initial insult or precipitating event, (2) the latent period, and (3) the chronic epilepsy phase.

The reactive astogliosis is an essential processes of epileptogenesis during the latent period that likely to be applicable to a wide spectrum of structural epilepsies and is a hallmark of the epileptic focus in humans and experimental models (Devinsky et al., 2013; Binder and Steinhäuser, 2021). Astrocytes are known to be involved in inflammation via their interactions with microglia and other innate immune cells along with the BBB dysfunction (Vezzani and Friedman, 2011; Devinsky et al., 2013; Vezzani et al., 2013; Ismail et al., 2021). The permeability of the BBB is altered in acquired epilepsies and this alteration occurs early after the epileptogenic injury as a result of extravasation of serum albumin and activation of TGb-Rs (Friedman et al., 2009). Albumin can bind transforming growth factor beta (TGF-β) receptors in astrocytes and leads a downregulation of Kir 4.1 and AQP4 channels, which facilitates neuronal hyperexcitability during epileptogenesis (Löscher and Friedman, 2020). Pharmacological blockade of TGb-RII signaling after status epilepticus in rodents rescued some of the molecular changes in astrocytes and reduced the incidence of epilepsy (Vezzani et al., 2022). The angiotensin II type 1 receptor antagonist, losartan, blocks brain TGF-β signaling and prevents epilepsy in animal models of epileptogenesis (Bar-Klein et al., 2014; Hong et al., 2019). Moreover, the interplay among the astrocytes, microglia and peripheral monocytes may lead to progression of epilepsy via increased leakage of the BBB and can affect epileptogenesis. Immune cells in the blood such as macrophages and monocytes are highly activated after seizures. Importantly, inhibiting the chemotaxis of leukocytes across the BBB reduces the severity of seizures. Early gabapentin treatment during the latency period successfully reduced microglial reactivity, neuroinflammation, and macrophage infiltration in Lithium-Pilocarpine model of epileptogenesis (Rossi et al., 2013, 2017).

HMGB-1 as an inflammatory cytokine released after the initial insult acting on astrocytes and microglia activates TLRs and its downstream signaling pathways, which are proposed as key early steps in epileptogenesis (Rosciszewski et al., 2019). Thus, either HMGB-1 blockage or TLR antagonist molecules would be able to reduce neuroinflammation and may be a novel target for developing anti-epileptogenic drugs.

Specific anti-epileptogenic therapies in structural epilepsies may also target gliotransmission (blockage of TNF-α driven astrocyte prunergic signaling; Nikolic et al., 2018; Vezzani et al., 2022), astrocytic gap junctions (Wallraff et al., 2006; Carlen, 2012; Volnova et al., 2022), neurotrophic factors (Vezzani et al., 2013; Araki et al., 2021), the glymphatic pathway (Eid et al., 2005; Binder et al., 2012; Tice et al., 2020), microglia-astrocyte-immune system communication (Rosciszewski et al., 2019), astroglial inflammatory pathways (Wetherington et al., 2008; Vezzani and Friedman, 2011; Ismail et al., 2021), the astrocytic Kir4.1-BDNF system (Kinboshi et al., 2020), astrocytic adenosine tone by using either adenosine receptor agonists or ADK inhibitors (Boison and Aronica, 2015), and DNA hypo- or hypermethylation of candidate genes in astrocytes (Williams-Karnesky et al., 2013).

In genetic generalized epilepsies astrocyte involvement during the epileptogenesis still limited. Current data indicate that, astrocytes get involved in disturbances of thalamic GABA-ergic and glutamatergic gliotransmission during the epileptogenesis that influences thalamocortical circuitry (Crunelli et al., 2020). Reactive astrogliosis with an increased GFAP expression within the thalamocortical loop is observed in genetic rat models of absence epilepsy (Dutuit et al., 2000; Çavdar et al., 2019). Cytokine (TNF-α, IL-1β) expression was associated with the development of absence seizures and tended to increase before the seizure onset in GAERS and WAG/Rij (Wistar Albino Glaxo rats from Rijswijk) rat models (Akin et al., 2011; van Luijtelaar et al., 2012). This is also in line with results showing that a specific anti-inflammatory approach, the interruption of the IL-6 signaling pathway through administration of tocilizumab, a neutralizing humanized monoclonal antibody to the IL-6 receptor reduced the development of absence seizures and depressive-like comorbidity in the GAERS model (Leo et al., 2020). In humans, valproic acid treatment lowers IL-6 serum levels in children with tonic–clonic generalized seizures (Steinborn et al., 2014). In order to treat this non-convulsive form of epilepsy and its associated neuropsychiatric comorbidities, targeting cytokines and chemokines may provide a new avenue for the development of targeted anti-epileptogenic therapies.

### 5.2. Role of astrocytes in ictogenesis and anti-seizure strategies

Ictogenesis is a fast, short-term electrical/chemical event that generate recurrent seizures. At the molecular level, ictogenesis involves excessive brain electrical discharges propagated by a cascade of chemical events initiated by the transmembrane voltage gated Na + channels with subsequent involvement of K^+^ channels and Ca^2+^ dependent release of neurotransmitters.

Recently, it was postulated that astrocytes play a significant role in ictogenesis. In structural epilepsies, astrocytes contribution in ictogenesis is relatively new area with limited data compared to what we know about epileptogenesis so far. However, growing evidence from experimental models and human studies suggests that reactive astrocytes play a key role in the initiation of seizures (Vezzani et al., 2022; Purnell et al., 2023). First clue of direct contribution of astrocytes to the generation of an epileptic discharge was the glutamate release from astrocytes promotes local synchronous activity in hippocampal neurons by acting on extrasynaptic NMDA receptors (Fellin et al., 2004). *In vitro* study showed that during epileptiform activity, the frequency of Ca^2+^ oscillations in astrocytes is dramatically increased and it is reduced by anti-seizure drugs valproate, gabapentin, and phenytoin (Tian et al., 2005) suggesting that their another mechanism of action is inhibition of gliotransmission. Purinergic receptor antagonists MRS2179 and MRS2500 (Alves et al., 2019) and the Panx1 channel inhibitors probenecid and mefloquine (Dossi et al., 2018) has been shown to inhibit Ca^2+^-mediated gliotransmission and have shown promise anti-seizure effect in experimental models. A study performed in *in vitro* model of focal seizures suggested that astrocytic Ca^2+^ elevation correlates with both the initial development and the maintenance of a ictal discharge but not interictal discharges, indicating that a recurrent excitatory loop (potentially including gliotransmission) between neurons and astrocytes increases seizure initiation and sustains the ictal discharge (Gómez-Gonzalo et al., 2010). In a recent study, optogenetic activation of astrocytes exhibited anti-seizure effect on neocortical seizures following cortical kainic acid injection, in a Na^+^/K^+^/ATPase dependent manner (Zhao et al., 2022). Authors investigated the anti-seizure mechanism of astrocyte stimulation and found that it was mediated by stimulation of the astrocytic Na^+^/K^+^/ATPase as a result of Na^+^ influx independent from Ca^2+^ signaling. These findings show that astrocytes can effectively control seizure initiation as well as seizure spread.

In addition to the astrocytic gliotransmission, other astrocytic dysfunctions such as loss of astrocytic gap junction coupling, as well as the impaired K^+^ clearance and glutamate homeostasis (Bedner et al., 2015), high levels of matrix metalloproteinases in astrocytes (Broekaart et al., 2021) are known to be involved in the initiation and progression of TLE.

So far, evidence showing the ictogenic involvement of astrocytes in genetic generalized epilepsies is still lacking. Observations from the rodent models of genetic absence epilepsy indicate that astrocyte modulation through inhibition of astrocytic acidic calcium-binding protein (S-100β) by a novel molecule ONO-2506 appeared to have specific anti-seizure properties in a genetic mouse model of absence epilepsy (Yamamura et al., 2013). The tonic GABA release of from astrocytes is one of the key mechanisms by which astrocytes play a key role in controlling absence seizures (Crunelli et al., 2011). Changes in GABAergic astrocyte-neuron signaling features in genetic rodent models of absence epilepsy may lead to the production of absence seizures. By modulating tonic GABA release from astrocytes, it might be possible to target absence seizures. Supporting this hypothesis putrescine which is a GABA precursor in astrocytes increased astrocytic GABA production and inhibited spontaneous SWDs in WAG/Rij rats (Kovács et al., 2022).

Despite these evidences, we are still far from understanding how astrocytes contribute to the generation of seizures, due to the many challenges in their investigation. First; astrocytes are diverse and have specific properties and functions in particular brain areas (Khakh and Deneen, 2019), second; reactive and compensatory phenotypes of astrocytes are not readily distinguishable and often coexist; third, difficult to distinguish the precise impact of astrocytes from that of neighboring neurons and fourth, individual astrocytes can even express both compensatory and pathological markers (Vezzani et al., 2022). Based on these facts we need selective tools as well as validated animal models to modulate astrocyte activity. Genetic approaches such as optogenetic, chemogenetic and Cre-technology have been largely used for selective activation or inhibition of astrocytes. Genetic mouse models generated by global and conditional gene deletion provide a platform for specifically targeting and manipulating astroctyes. There are several mouse lines, in which cre-lox system was used to conditionally delete genes in astrocytes by coupling cre with other reporters such as GFAP reporter, GLAST, or GLT-1 (Srinivasan et al., 2016). Another novel and promising alternative approach is to use viral vectors for gene delivery to astrocytes in specific brain regions (Nagai et al., 2019). Given the substantial insights made over the past decade, glial cells are attractive targets for new drug development for neurological disorders. However, there is an important challenge of delivering drugs across the BBB for targeting glial cells. Fortunately, new developments in drug delivery research can now address this challenge by describing synthetic nanoparticles that can be used for astrocyte-specific targeting, as well as developing non-invasive molecular delivery strategies that bypass the BBB including receptor-mediated transcytosis, neurotrophic viruses, and exosomes (Terstappen et al., 2021; Lee et al., 2022). All these abovementioned factors can help to understand the precise pathways by which astrocytes (and probably other glial cells) contribute to epileptogenesis and ictogenesis, and design novel therapeutic approaches that can be translated into the clinic.

## Author contributions

NÇ: literature review, writing the manuscript, and drawing the figures. FO and ES: literature review and writing and editing the manuscript. All authors contributed to the article and approved the submitted version.

## Funding

Research in the Onat’s lab was supported by European Commission Horizon Europe Program under the call HORIZON-WIDERA-2021-ACCESS-03 (Grant Number 101078981-GEMSTONE).

## Conflict of interest

The authors declare that the research was conducted in the absence of any commercial or financial relationships that could be construed as a potential conflict of interest.

## Publisher’s note

All claims expressed in this article are solely those of the authors and do not necessarily represent those of their affiliated organizations, or those of the publisher, the editors and the reviewers. Any product that may be evaluated in this article, or claim that may be made by its manufacturer, is not guaranteed or endorsed by the publisher.
